# Supplementary material for: The Relationships between Prospection, Self-Efficacy, and Depression in College Students with Cross-Lagged Analysis
Source: Int J Environ Res Public Health. 2022 Nov 9;19(22):14685. doi: 10.3390/ijerph192214685 (PMC9690034; doi:10.3390/ijerph192214685)
Supplement: Supplementary file 1 [file ijerph-19-14685-s001.zip › ijerph-1993099-supplementary.pdf]

Table S1. *The SCEFT performance at T1 and T2 in valid participants (N = 276).*

	**T1**<br>**Mean (SD)**	**T2**<br>**Mean (SD)**
**Specificity**		
Specific events	0.23(0.14)	0.23(0.15)
Extended events	0.27(0.15)	0.27(0.16)
Categoric events	0.22(0.15)	0.19(0.13)
Sematic associations	0.26(0.16)	0.28(0.17)
Omissions	0.02(0.05)	0.03(0.06)
**Emotion**		
Positive	0.51(0.21)	0.50(0.20)
Neutral	0.44(0.19)	0.43(0.18)
Negative	0.03(0.06)	0.04(0.07)

The SCEFT, the Sentence Completion for Events in the Future Test; T1, Time point 1; T2, time point 2
